# Potential ocular indicators to distinguish posterior cortical atrophy and typical Alzheimer’s disease: a cross-section study using optical coherence tomography angiography

**DOI:** 10.1186/s13195-024-01431-w

**Published:** 2024-03-25

**Authors:** Yan Sun, Lumi Zhang, Hui Ye, Lumin Leng, Yi Chen, Yujie Su, Peifang Ren, Hong Lu, Guoping Peng

**Affiliations:** 1 Department of Neurology, the First Affiliated Hospital, Zhejiang University School of Medicine, Hangzhou, People’s Republic of China; 2 Department of Ophthalmology, the First Affiliated Hospital, Zhejiang University School of Medicine, Hangzhou, People’s Republic of China

**Keywords:** Posterior cortical atrophy, Alzheimer’s disease, Optical coherence tomography, Optical coherence tomography angiography, Ocular fundus structure, Retinal vascular plexus, Early diagnosis

## Abstract

**Background:**

Posterior cortical atrophy (PCA) is a form of dementia that frequently displays significant visual dysfunction and relatively preserved cognitive and executive functions, thus hindering early diagnosis and treatment. This study aimed to investigate possible fundus markers in PCA patients and compare them with those of typical Alzheimer’s disease (AD) patients to seek potential diagnostic patterns.

**Methods:**

Age-matched PCA and AD patients and healthy controls (HC) completed optometry, intraocular pressure measurement, neuropsychologic assessments, optical coherence tomography (OCT), and optical coherence tomography angiography (OCTA) examination in one visit. Overall, six outcomes of thicknesses of various retinal layers and seven outcomes of the retinal microvascular network were calculated. After adjusting for age, sex, and years of education, the OCT and OCTA results were analyzed using analysis of covariance and generalized linear models. Correlation analyses were performed using Spearman correlation, and ROC curves were plotted.

**Results:**

Twelve PCA patients, nineteen AD patients, and thirty HC, aged 45–80 years were included. Fifty HC, thirty AD, and twenty PCA eyes were available for foveal avascular zone (FAZ) area analysis; forty-nine HC, thirty-four AD, and eighteen PCA eyes were available for OCT and OCTA assessments. PCA patients had thinner retinal nerve fiber layer and ganglion cell layer + inner plexiform layer than HC in the 0–3 mm circle and 1–3 mm ring. Few structural differences were observed between the AD group and the other two groups. The flow area of the superficial capillary plexus and the intermediate capillary plexus was smaller in the PCA group than in the HC group in the 0–1 mm circle, 0–3 mm circle. MMSE performed better than any combination of optical parameters in identifying AD and PCA from HC (AUC = 1), while the combination of MoCA, retinal thickness and vascular density of ICP in the 1-3 mm ring, with flow area of ICP in the 0-1 mm circle showed the strongest ability to distinguish PCA from AD (AUC = 0.944).

**Conclusions:**

PCA patients exhibited similar impairment patterns to AD patients in the fundus structure and microvascular network. OCTA may aid in the non-invasive detection of AD and PCA, but still remains to be substantiated.

**Supplementary Information:**

The online version contains supplementary material available at 10.1186/s13195-024-01431-w.

## Background

Posterior cortical atrophy (PCA) is a neurodegenerative disease characterized by a gradual and selective functional impairment and structural alternation of the parietal and occipital lobes, with an early onset between 50 and 65 years. Visual impairment and cognitive failure associated with parieto-occipital cortical atrophy such as dysmetria, alexia, and ignorance are the main symptoms in PCA patients, while the routine ophthalmic clinical examination frequently cannot find obvious abnormalities of ocular optic structures [1, 2]. Although PCA patients frequently share similar pathologic characteristic with Alzheimer’s disease (AD), they can exhibit different patterns of clinical manifestations and structural and/or functional neuroimaging [3]. Currently, the auxiliary examination techniques for PCA include magnetic resonance imaging, single photon emission computed tomography, and positron emission computed tomography, as well as cerebrospinal fluid, which may be inaccurate, invasive, or expensive. The above-mentioned condition combined with the confusing nature of the non-amnesic symptoms and the relatively low prevalence (probably underestimated owing to the lack of awareness of PCA) make it more difficult for an early diagnosis and intervention in PCA.

The retina has the same embryonic origin as some structures in the central nervous system (CNS); the blood supply to the retina is derived from the capillary network of the CNS and the retina has neurons, astrocytes, and microglia that are closely connected with the CNS [4, 5]. Therefore, retinal and choroidal examinations have offered a new approach to investigate the development of neurologic diseases in recent decades. A co-atrophy pattern of the entire cerebral volume or a particular brain region and retinal thickness has been reported in the normal aging population [6, 7] together with a variety of neurological diseases, including mild cognitive impairment [8], AD [9, 10], Parkinson's disease [6], and multiple sclerosis [11] among others.

Optical coherence tomography (OCT) and optical coherence tomography angiography (OCTA) are non-invasive techniques to evaluate the structure and choroidal system of the ophthalmic fundus respectively. Compared with traditional angiography techniques such as fluorescein fundus angiography and indocyanine green angiography, OCTA images provide higher contrast and clarity without the use of any dye. Furthermore, individually visualizing retinal and choroidal capillaries in separate layers, as well as capillary pathology, is practicable using special analytical processing software. With the progress in OCT and OCTA technologies, swept-source optical coherence tomography (SS-OCT) and OCTA have provided greater scanning speed and penetration, making the analysis of choroidal structures more convenient [12, 13].

The results from earlier studies of optic fundus structure and blood flow in PCA patients were insufficient for the limited number of relative researches, and those in AD patients were not substantiated. Retinal nerve fiber layer (RNFL) thinning was observed in many cross-sectional [14, 15] and prospective studies [16, 17]. However, den Haan et al. indicated that RNFL cannot be used as an ocular marker to distinguish PCA or typical AD from normal controls [18]. Strongly debated conclusions have also been drawn in studies regarding vascular indicators such as the foveal avascular zone (FAZ) area [19–21].

In clinical practice, it is still difficult to distinguish PCA from classic AD purely from clinical manifestations and neuropsychological assessment, especially for patients in the middle or advanced stages of the both diseases. Owning to the significantly decreased visual perception ability, exploring the change of fundus markers in PCA patients and typical AD patients may provide a new direction for clinical diagnosis of PCA. Therefore, we further investigated and compared the patterns of changes in fundus structure and perfusion in PCA and typical AD patients by the SS-OCT and OCTA techniques to find possible indicators that can screen out those patients with PCA or AD at an early point.

## Methods

### Study participants

All the PCA patients, AD patients, and healthy controls (HC) were enrolled in the First Affiliated Hospital, Zhejiang University School of Medicine from August 3, 2021 to August 6, 2022. Patients with PCA were required to meet the core features of "PCA clinico-radiological syndrome (classification level 1)" in the "Consensus classification of posterior cortical atrophy" proposed by Crutch et al [22]. Patients with AD met the core clinical criteria of probable AD (with or without AD pathophysiologic process) proposed by the National Institute on Aging and the Alzheimer’s Association in 2011 [23]. Some of the HC were family members who accompanied PCA or AD patients to the clinic, and the other portion were age-matched individuals who are healthy volunteers without cognitive impairment complaints and suffered no severe organic disease.

The age range was 45–80 years and the patients had the ability to cooperate with the performance of ophthalmic examinations (optometry and intraocular pressure measurements), SS-OCT/OCTA, and neuropsychologic assessments (Mini-mental State Examination (MMSE), Montreal Cognitive Assessment (MoCA) and clinical dementia rating scale (CDR). Registration and Recall parts (maximum score for 6) in MMSE were used for memory assessment, as sentence writing and picture copying parts (maximum score for 2) for visuospatial ability assessment. The participants were excluded with: obesity, diabetes, poorly controlled blood pressure (hypertension or hypotension), ocular diseases (e.g., glaucoma, capillary retinopathy, age-related macular deformation, severe cataract), cerebrovascular diseases (e.g., cerebral hemorrhage, intracranial arteritis, inflammatory vasculitis of the central nervous system, Moyamoya disease), an eye pressure > 21 mmHg, sphere >  − 6.00 D or 2.00 D, and/or cylinder > 3.00 D in the ophthalmologic examination.

### OCT and OCTA scanning protocol

**Fig. 1:**
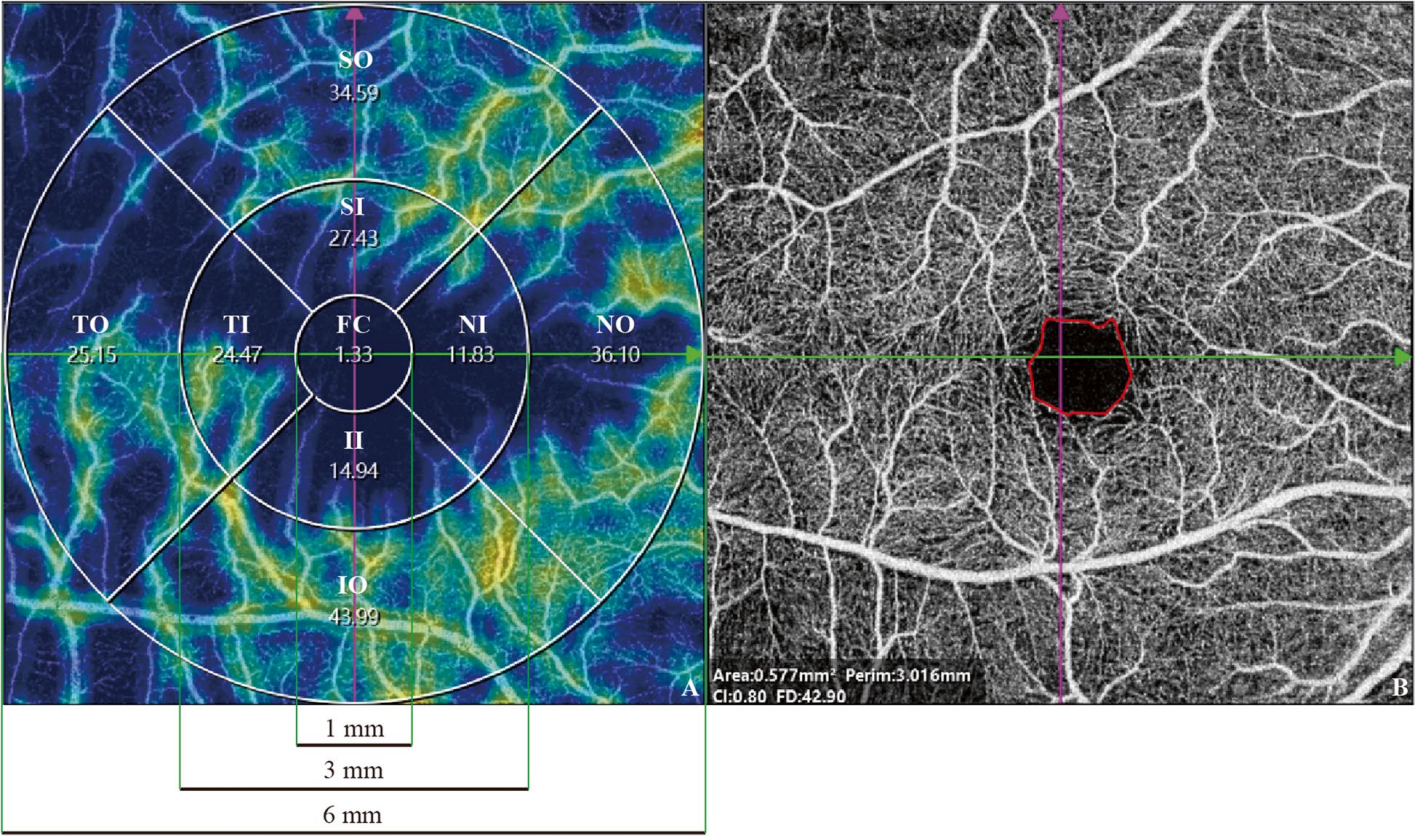
Illustrations for SS-OCT and OCTA image quantification. **A** The ETDRS grid of one participant’s right eye: the ETDRS grid, centered on the fovea of the macula, divides the retina into nine regions with three rings and four quadrants. FC for foveal center; SI for superior inner; NI for nasal inner; II for inferior inner; TI for temporal inner; SO for superior outer; NO for nasal outer; IO for inferior outer; and TO for temporal outer. Pericentral ring refers to the concentric ring from 1 to 3 mm diameter (i.e., SI + NI + II + TI). Peripheral ring refers to the concentric ring from 3 to 6 mm diameter (i.e., SO + NO + IO + TO). **B** FAZ quantification image of one participant’s right eye, marked by the red curve

SS-OCT and SS-OCTA images were obtained using an SS-OCT system (VG200, SVision Imaging, Ltd., Luoyang, China) with a tunable laser of 1050 nm wavelength and a scanning speed of 200,000 A-scan per second. The axial optical resolution of SS-OCT was 5 μm, the lateral resolution was 13 μm, and the scanning depth was 3 mm. The axial digital resolution was 2.7 mm/1024 pixels. SS-OCT and OCTA images, presented as the Early Treatment Diabetic Retinopathy Study (ETDRS) grid, were collected in 6 mm × 6 mm, 512 × 512 pixels, R4, with each B-scan consisting of 512 A-scans, while the FAZ area was measured in 3 mm × 3 mm (Fig. 1).

The system was equipped with an eye-tracking tool based on an integrated confocal scanning laser ophthalmoscope which could eliminate eye-movement artifacts. Eye images with a signal strength < 7 were excluded in the study, which was measured during the scanning in real time with the processing software version 2.1.016 [24]. All eye images were examined by two ophthalmologists (PFR and HY) to exclude conditions that could have affected follow-up data measurements and analysis.

### Optical parameters

For fundus structures, the software automatically segmented and measured the thickness of the choroid, retina, ganglion cell layer (GCL) + inner plexiform layer (IPL), inner nuclear layer (INL), RNFL, and GCL + IPL + RNFL, for a total of six outcomes. The superficial capillary plexus (SCP) was the vascular complex between the inner boundary of the internal limiting membrane and the outer boundary of the GCL; the intermediate capillary plexus (ICP) was, in principal, located at the inner side of the INL bordering the IPL; and the deep capillary plexus (DCP) was located at the outer side of the INL bordering the outer plexiform layer. The flow area and capillary density were respectively calculated in the SCP, ICP, and DCP after automatic segmentation by built-in algorithms. Additionally, measurement of the area, perimeter, circularity index, and fractal dimension of the FAZ were conducted using the SS-OCTA tool.

### Statistical analysis

Quantitative variables were expressed as mean ± standard deviation or median with 95% confidence interval. Demographic and clinical data were analyzed using one-way analysis of variance or Kruskal–Wallis test for quantitative data and chi-square test for qualitative data. Bonferroni method was applied for post-hoc multiple comparisons. Age and years of education, which spanned a wide range in the participants’ demographic data, and sex, which had an unbalanced composition ratio between the three groups, were used as covariates. For data about fundus structure and capillary perfusion, after adjusting for the covariates, comparisons were made using analysis of covariance when conforming to a normal distribution with homogeneous variance or using generalized linear models when not conforming to a normal distribution or homogeneous variance. Analysis of the correlation of capillary and fundus structure with cognition was conducted via Spearman's correlation. The optimal diagnostic models were determined using multivariate Logistic regression. Receiver operating characteristic curve (ROC) analyses presented the accuracy of the OCT and OCTA data in distinguishing between PCA, AD, and HC. The diagnostic efficiency was appraised by the area under a ROC (AUC). A *p*-value < 0.05 was considered to be significant. All the statistical analyses and figure generation were completed in IBM SPSS Statistics 25 and GraphPad Prism 9.

## Results

### Demographic and clinical data

In total, 12 PCA patients, 19 AD patients, and 30 HC were included in the study. There was no significant difference between the three groups in demographic data, including age, sex proportion, and years of education, as well as routine optical examination results, such as sphere, cylinder, axial, and intraocular pressure (all *p* > 0.05). The PCA and AD groups had lower MMSE and MoCA scores than the HC group (all *p* < 0.001), and the PCA group displayed a lower MoCA score than the AD group (8.75 ± 4.48 vs 13.21 ± 5.79, *p* < 0.05). CDR scores showed no difference between the AD and PCA groups (1.79 ± 0.86 vs 2.00 ± 0.60, *p* = 0.655) (Table 1).

**Table 1:** Demographical and clinical data of participants

Characteristics	HC	AD	PCA	*P*-value
	(n = 30)	(n = 19)	(n = 12)	
Age (years)	61.93 ± 5.11	62.53 ± 6.96	62.42 ± 5.73	0.934
Gender (M/F)	9/21	10/9	7/5	0.139
Education (years)	9.00 ± 3.95	8.53 ± 4.16	9.83 ± 4.45	0.691
MMSE	27.40 ± 1.85	17.32 ± 6.56^*^	12.92 ± 4.54^*^	** < 0.001**
Memory^a^	5.10 ± 0.96	3.00 ± 1.37^*^	3.08 ± 1.00^*^	** < 0.001**
Visuospatial ability^b^	1.90 ± 0.31	1.16 ± 0.83^*^	0.17 ± 0.39^*^	** < 0.001**
MoCA	23.73 ± 3.33	13.21 ± 5.79^*^	8.75 ± 4.48^*#^	** < 0.001**
CDR	0	1.79 ± 0.86^*^	2.00 ± 0.60^*^	** < 0.001**
SPH (D)	-0.15 ± 1.11	-0.42 ± 1.40	-0.41 ± 1.21	0.577
CYL (D)	0.86 ± 0.63	0.61 ± 0.56	0.89 ± 0.95	0.178
AX (degree)	123.04 ± 73.10	122.35 ± 70.87	131.75 ± 68.49	0.896
IOP (mmHg)	14.65 ± 2.61	13.51 ± 3.03	13.27 ± 3.10	0.053

*Abbreviations*: *HC* Healthy control, *AD* Alzheimer’s disease, *PCA* Posterior cortical atrophy, *MMSE* Mini-Mental State Examination, *MoCA* Montreal Cognitive Assessment, *CDR* Clinical dementia rating scale, *SPH* Sphere, *CYL* cylinder, *AX* Axial, *IOP* Intra-ocular pressure. Assessment values are mean ± standard deviation (SD), or n. The bold values indicate statistical significance

^*^ *p* < 0.001: AD, PCA vs HC, by post hoc test

^#^ *p* < 0.05: AD vs PCA, by post hoc test

^a^ Memory refers to Registration and Recall parts in MMSE, maximum score for 6

^b^ Visuospatial ability refers to sentence writing and picture copying parts in MMSE, maximum score for 2

Following evaluation by two ophthalmologists, eyes with low image quality and/or ocular diseases that met the exclusion criteria were excluded. Finally, 50 eyes of HC (oculus dexter (OD) *n* = 25, oculus sinister (OS) *n* = 25), 30 eyes of AD (OD *n* = 16, OS *n* = 14), and 20 eyes of PCA patients (OD *n* = 9, OS *n* = 11) were available in the FAZ analysis; while 49 eyes of HC (OD *n* = 26, OS *n* = 23), 34 eyes of AD (OD *n* = 17, OS *n* = 17) and 18 eyes of PCA patients (OD *n* = 8, OS *n* = 10) were available for the SS-OCT and OCTA assessments. Because of high eccentricity in most eyes, we aborted the data of the peripheral circle (i.e., regions from 3 to 6 mm in the ETDRS grid) in the subsequent statistical analysis to assure the accuracy of the results. All optical parameters were compared between three groups in OD separately, OS separately, and OD mixing OS.

### Fundus structural differences assessed by SS-OCT

The thickness of six separate or combined layers of the fundus were compared between the three groups in seven regions (0–1 mm circle, 0–3 mm circle, 1–3 mm ring, superior inner (SI), temporal inner (TI), inferior inner (II), nasal inner (NI) (Fig. 1A)).

Choroidal thickness was greater in the PCA group than in the AD group in the 0–1 mm circle (353.67 ± 89.50 vs 289.91 ± 89.59 μm, *p* = 0.040) and TI (349.86 ± 85.13 vs 285.11 ± 83.72 μm, *p* = 0.024). No differences were observed between the HC group and the PCA or AD groups (both *p* > 0.05) (Supplementary Table 1).

Retinal thickness was less in the PCA group than in the HC group in the 0–3 mm circle (315.97 ± 12.32 vs 321.29 ± 13.32 μm, *p* = 0.021) and NI (326.32 ± 14.63 vs 335.39 ± 14.25 μm, *p* = 0.003) (Supplementary Table 2). GCL + IPL thickness was less in the PCA group than in the HC group in the 0–3 mm circle (76.91 ± 9.17 vs 79.56 ± 5.62 μm, *p* = 0.003), 1–3 mm ring (83.69 ± 9.72 vs 86.54 ± 5.90 μm, *p* = 0.008), SI (85.02 ± 8.31 vs 88.07 ± 5.96 μm, *p* = 0.018), and NI (83.47 ± 12.13 vs 87.69 ± 6.34 μm, *p* = 0.0201) (Supplementary Table 3). INL thickness was less in the PCA group than in the HC group in NI (45.38 ± 2.84 vs 47.07 ± 4.06 μm, *p* = 0.035) (Supplementary Table 4). RNFL thickness was less in the PCA group than in the HC group in the 0–3 mm circle (23.86 ± 3.40 vs 24.66 ± 2.21 μm, *p* = 0.034), 1–3 mm ring (25.21 ± 3.77 vs 26.06 ± 2.77 μm, *p* = 0.041), and NI (23.68 ± 3.77 vs 25.03 ± 2.86 μm, *p* = 0.008) (Supplementary Table 5). No difference was observed in the GCL + IPL + RNFL thickness (*p* > 0.05) (Supplementary Table 6). The AD group presented no significant difference with the HC and PCA groups in all five structural thicknesses (all *p* > 0.05).

### Fundus capillary differences assessed by SS-OCTA

Capillary density and flow area of the SCP, ICP, and DCP, six outcomes in total, were compared between the three groups in the same seven regions (0–1 mm circle, 0–3 mm circle, 1–3 mm ring, SI, TI, II, NI).

**Fig. 2:**
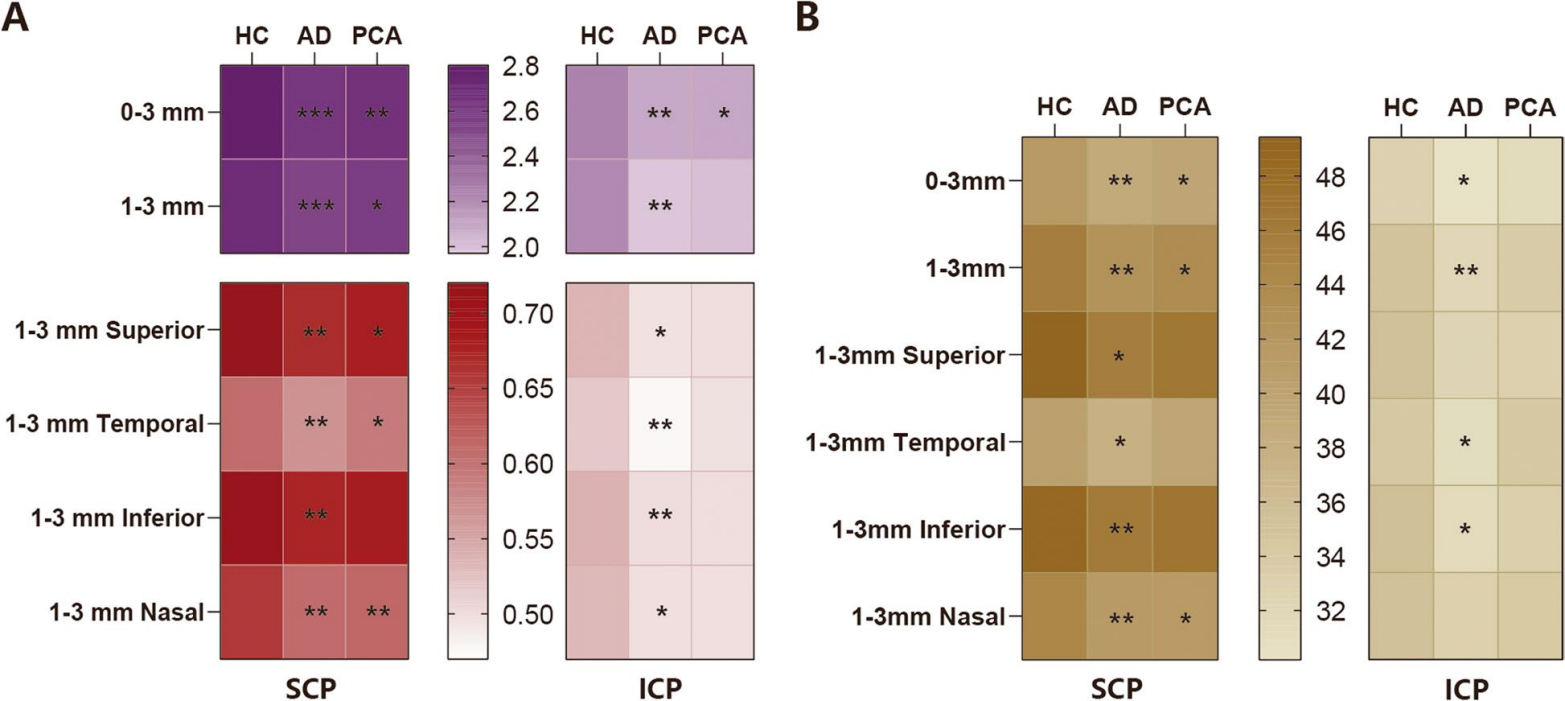
Heat maps showing the difference of mean level of flow area and capillary density. **A** Flow area (mm^2^) in the superficial capillary plexus (SCP) (left) and the intermediate capillary plexus (ICP) (right). **B** Capillary density (%) in the SCP (left) and ICP (right). AD, PCA vs HC: ^*^ *p* < 0.05, ^**^ *p* < 0.01, ^***^ *p* < 0.001

For the capillary density of the SCP, this was lower in the AD group than in the HC group in the 0–3 mm circle (*p* = 0.001), 1–3 mm ring (*p* = 0.001), SI (*p* = 0.002), TI (*p* = 0.019), II (*p* = 0.009), and NI (*p* = 0.007); while it was lower in the PCA group than in the HC group in the 0–1 mm circle (*p* = 0.008), 0–3 mm circle (*p* = 0.024), 1–3 mm ring (*p* = 0.048), and NI (*p* = 0.020) (Table 2 and Fig. 2). For the capillary density of the ICP, this was lower in the AD group than in the HC group in the 0–3 mm circle (*p* = 0.020), 1–3 mm ring (*p* = 0.008), TI (*p* = 0.046), and II (*p* = 0.013); while it was lower in the PCA group than in the AD group in the 0–1 mm circle (*p* = 0.044) (Table 2 and Fig. 2). There was no significant difference in the capillary density of the DCP between the three groups in any quadrant (all *p* > 0.05) (Supplementary Table 7).

**Table 2:** Comparison of flow area and vascular density of ICP and SCP in three groups

	**HC (n = 49)**	**AD (n = 34)**	**PCA (n = 18)**
**Flow area of ICP (mm**^**2**^**)**			
0–1 mm circle	0.1186 ± 0.0321	0.1197 ± 0.0448	0.1025 ± 0.0221^**^
0–3 mm circle	2.2454 ± 0.1589	2.0904 ± 0.2746^**^	2.0949 ± 0.2319^*^
1–3 mm ring	2.2167 ± 0.1567	1.9707 ± 0.2487^**^	1.9923 ± 0.2248
Superior inner	0.5370 ± 0.0417	0.4962 ± 0.0862^*^	0.4987 ± 0.0658
Temporal inner	0.5179 ± 0.0481	0.4763 ± 0.0686^**^	0.4966 ± 0.0551
Inferior inner	0.5382 ± 0.0454	0.5000 ± 0.0602^**^	0.4994 ± 0.0571
Nasal inner	0.5336 ± 0.0417	0.4982 ± 0.0766^*^	0.4976 ± 0.0642
**Vascular density of ICP (%)**			
0–1 mm circle	14.3594 ± 4.7597	15.4557 ± 7.3033	12.1258 ± 2.9380^†^
0–3 mm circle	32.8780 ± 3.4920	30.1879 ± 5.4381^*^	31.2835 ± 3.7517
1–3 mm ring	35.1994 ± 3.7448	32.0346 ± 5.5376^**^	33.6852 ± 4.1439
Superior inner	35.4247 ± 4.5063	32.4111 ± 7.6077	32.9766 ± 5.7374
Temporal inner	34.3944 ± 5.1393	31.0571 ± 6.5958^*^	34.3877 ± 4.7689
Inferior inner	35.5829 ± 4.8639	31.5183 ± 6.9000^*^	33.2461 ± 4.7481
Nasal inner	35.3952 ± 4.1250	33.1594 ± 8.1473	34.1256 ± 5.4461
**Flow area of SCP (mm**^**2**^**)**			
0–1 mm circle	0.0907 ± 0.0330	0.0949 ± 0.0460	0.0771 ± 0.0212^** †^
0–3 mm circle	2.7850 ± 0.2063	2.6128 ± 0.3225^***^	2.6443 ± 0.3781^**^
1–3 mm ring	2.6942 ± 0.1964	2.5180 ± 0.3004^***^	2.5672 ± 0.3670^*^
Superior inner	0.7198 ± 0.0550	0.6674 ± 0.1024^**^	0.6794 ± 0.0905^*^
Temporal inner	0.6064 ± 0.0483	0.5687 ± 0.0797^**^	0.5921 ± 0.0833
Inferior inner	0.7122 ± 0.0611	0.6733 ± 0.0687^**^	0.6818 ± 0.1052^*^
Nasal inner	0.6559 ± 0.0537	0.6085 ± 0.0897^**^	0.6139 ± 0.1032^**^
**Vascular density of SCP (%)**			
0–1 mm circle	8.9057 ± 4.2500	8.9495 ± 5.8354	7.2404 ± 2.8073^**^
0–3 mm circle	41.6584 ± 3.4751	38.9816 ± 5.1625^**^	39.6129 ± 7.1054^*^
1–3 mm ring	45.7641 ± 3.7437	42.7460 ± 5.4349^**^	43.6711 ± 7.7809^*^
Superior inner	49.4141 ± 3.9880	45.7721 ± 7.3140^*^	46.7333 ± 7.7789
Temporal inner	40.3776 ± 2.8981	37.8033 ± 6.2844^*^	39.5759 ± 7.0591
Inferior inner	48.7654 ± 4.6209	46.0670 ± 5.0531^**^	46.9004 ± 8.5887
Nasal inner	44.5076 ± 4.4180	41.3367 ± 6.8787^**^	41.4901 ± 6.8445^*^

*Abbreviations*: *HC* Healthy control, *AD* Alzheimer’s disease, *PCA* Posterior cortical atrophy, *ICP* Intermediate capillary plexus, *SCP* Superior capillary plexus. Assessment Values are mean ± standard deviation (SD)

^*^ *p* < 0.05: AD, PCA vs HC, by post hoc test

^**^ *p* < 0.01: AD, PCA vs HC, by post hoc test

^***^ *p* < 0.001: AD, PCA vs HC, by post hoc test

^†^ *p* < 0.05: AD vs PCA, by post hoc test

Regarding the flow area of the SCP, this was smaller in the AD group than in the HC group in the 0–3 mm circle (*p* < 0.001), 1–3 mm ring (*p* < 0.001), SI (*p* = 0.001), TI (*p* = 0.005), II (*p* = 0.004), and NI (*p* = 0.001); while this was smaller in the PCA group than in the AD group in the 0–1 mm circle (*p* = 0.030) and smaller than in the HC group in the 0–1 mm circle (*p* = 0.004), 0–3 mm circle (*p* = 0.009), 1–3 mm ring (*p* = 0.024), SI (*p* = 0.045), II (*p* = 0.040), and NI (*p* = 0.009) (Table 2 and Fig. 2). The flow area of the ICP was smaller in the AD group than in the HC group in the 0–3 mm circle (*p* = 0.002), 1–3 mm ring (*p* = 0.002), SI (*p* = 0.010), TI (*p* = 0.007), II (*p* = 0.007), and NI (*p* = 0.021); while it was smaller in the PCA group than in the HC group in the 0–1 mm circle (*p* = 0.009) and 0–3 mm circle (*p* = 0.027) (Table 2 and Fig. 2). For the flow area of the DCP, only that of the TI area of the AD group was significantly smaller than in the HC group (0.3263 ± 0.0867 vs 0.3685 ± 0.0537 mm^2^, *p* = 0.011) (Supplementary Table 8).

### FAZ assessment

There was no significant difference between the PCA, AD, and HC groups in the FAZ area (0.3948 ± 0.1239 vs 0.3995 ± 0.1278 vs 0.4144 ± 0.1094 mm^2^, *p* = 0.769), perimeter (2.622 ± 0.4205 vs 2.684 ± 0.3852 vs 2.718 ± 0.3679 mm, *p* = 0.641), circularity index (0.7064 ± 0.0578 vs 0.6805 ± 0.0779 vs 0.6969 ± 0.0736, *p* = 0.424), or fractal dimension (40.50 ± 4.60 vs 39.54 ± 4.34 vs 41.49 ± 3.44, *p* = 0.106) (Supplementary Table 9).

### Correlation analysis of optic outcomes and cognitive state

To verify the matching of different OCTA data in one region, the correlation analysis between the capillary density and flow area in the ICP and SCP was conducted, showing a high correlation in all seven quadrants (*r* ranged from 0.8359 to 0.8707 for the ICP and from 0.9110 to 0.9723 for the SCP, all *p* < 0.0001).

On the basis of the analysis results above, indicators that differed significantly between groups were selected for further correlation analysis. The capillary density and flow area of the SCP was of weak to moderate correlation with the RNFL thickness, and of moderate to high correlation with the GCL + IPL in all seven quadrants (Table 3), whereas the capillary density and flow area of the ICP only displayed a strong correlation with the GCL + IPL in the 0–1 mm circle (*r* = 0.6439, *r* = 0.7848, respectively, both *p* < 0.0001) and a moderate to strong correlation with the INL in the 0–1 mm circle (*r* = 0.4645, *r* = 0.6588, respectively, both *p* < 0.0001).

**Table 3:** Correlation between capillary density, flow area of SCP and thickness of RNFL, GCL + IPL

	**Capillary density**		**Flow area**	
	*r*	*p*	*r*	*p*
**RNFL**				
0–1 mm circle	0.3870	** < 0.0001**	0.4190	** < 0.0001**
0–3 mm circle	0.4859	** < 0.0001**	0.4573	** < 0.0001**
1–3 mm ring	0.4657	** < 0.0001**	0.4348	** < 0.0001**
Superior inner	0.4977	** < 0.0001**	0.4569	** < 0.0001**
Temporal inner	0.3113	**0.0015**	0.2805	**0.0045**
Inferior inner	0.5187	** < 0.0001**	0.4892	** < 0.0001**
Nasal inner	0.4934	** < 0.0001**	0.5039	** < 0.0001**
**GCL + IPL**				
0–1 mm circle	0.8498	** < 0.0001**	0.8885	** < 0.0001**
0–3 mm circle	0.5975	** < 0.0001**	0.5948	** < 0.0001**
1–3 mm ring	0.5855	** < 0.0001**	0.5749	** < 0.0001**
Superior inner	0.5919	** < 0.0001**	0.6171	** < 0.0001**
Temporal inner	0.4716	** < 0.0001**	0.4490	** < 0.0001**
Inferior inner	0.6134	** < 0.0001**	0.6081	** < 0.0001**
Nasal inner	0.6067	** < 0.0001**	0.6176	** < 0.0001**

*Abbreviation*: *SCP* Superficial capillary plexus, *RNFL* Retinal nerve fiber layer, *GCL* Ganglion cell layer, *IPL* Inner plexus layer. Bold type of *p* for statistical significance

A weak correlation of vessel density and perfusion area with the MMSE and MoCA scores was found in some quadrants of the SCP and ICP in all participants (Table 4). For subdomains in MMSE, only 0–3 mm circle, 1–3 mm ring, and II areas of ICP showed a weak correlation with visuospatial ability (Supplementary Table 10).

**Table 4:** Correlation between capillary density, flow area of SCP and ICP with MMSE and MoCA score

	**SCP**				**ICP**			
	**Vessel density**		**Flow area**		**Vessel density**		**Flow area**	
	*r*	*p*	*r*	*p*	*r*	*p*	*r*	*p*
**MMSE**								
0–1 mm circle	-0.0003	0.9975	-0.0710	0.4806	-0.0504	0.6170	-0.0386	0.7015
0–3 mm circle	0.1637	0.1020	0.1874	0.0606	0.1895	0.0576	0.2662	**0.0071**
1–3 mm ring	0.1602	0.1095	0.1974	**0.0479**	0.2116	**0.0337**	0.2847	**0.0039**
Superior inner	0.1802	0.0713	0.2009	**0.0439**	0.2024	**0.0423**	0.2562	**0.0097**
Temporal inner	0.0654	0.5157	0.0970	0.3344	0.1040	0.3007	0.1932	0.0529
Inferior inner	0.1116	0.2667	0.1417	0.1575	0.3144	**0.0014**	0.3068	**0.0018**
Nasal inner	0.2254	**0.0234**	0.2443	**0.0138**	0.1134	0.2587	0.2248	**0.0238**
**MoCA**								
0–1 mm circle	0.0209	0.8357	-0.0526	0.6013	-0.0214	0.8319	-0.0333	0.7411
0–3 mm circle	0.1672	0.0948	0.1826	0.0676	0.1947	0.0511	0.2557	**0.0098**
1–3 mm ring	0.1631	0.1031	0.1909	0.0558	0.2119	**0.0334**	0.2771	**0.0050**
Superior inner	0.1822	0.0683	0.1943	0.0515	0.1879	0.0599	0.2307	**0.0203**
Temporal inner	0.0398	0.6924	0.0669	0.5063	0.0983	0.3280	0.1835	0.0663
Inferior inner	0.1290	0.1984	0.1611	0.1076	0.3272	**0.0008**	0.3142	**0.0014**
Nasal inner	0.2291	**0.0212**	0.2349	**0.0181**	0.1257	0.2102	0.2325	**0.0193**

*Abbreviation*: *SCP* superficial capillary plexus, *MMSE* Mini-mental State Examination, *MoCA* Montreal Cognitive Assessment. Bold type of *p* for statistical significance

### ROC curves of logistic regression models in distinguishing PCA, AD, and HC

**Fig. 3:**
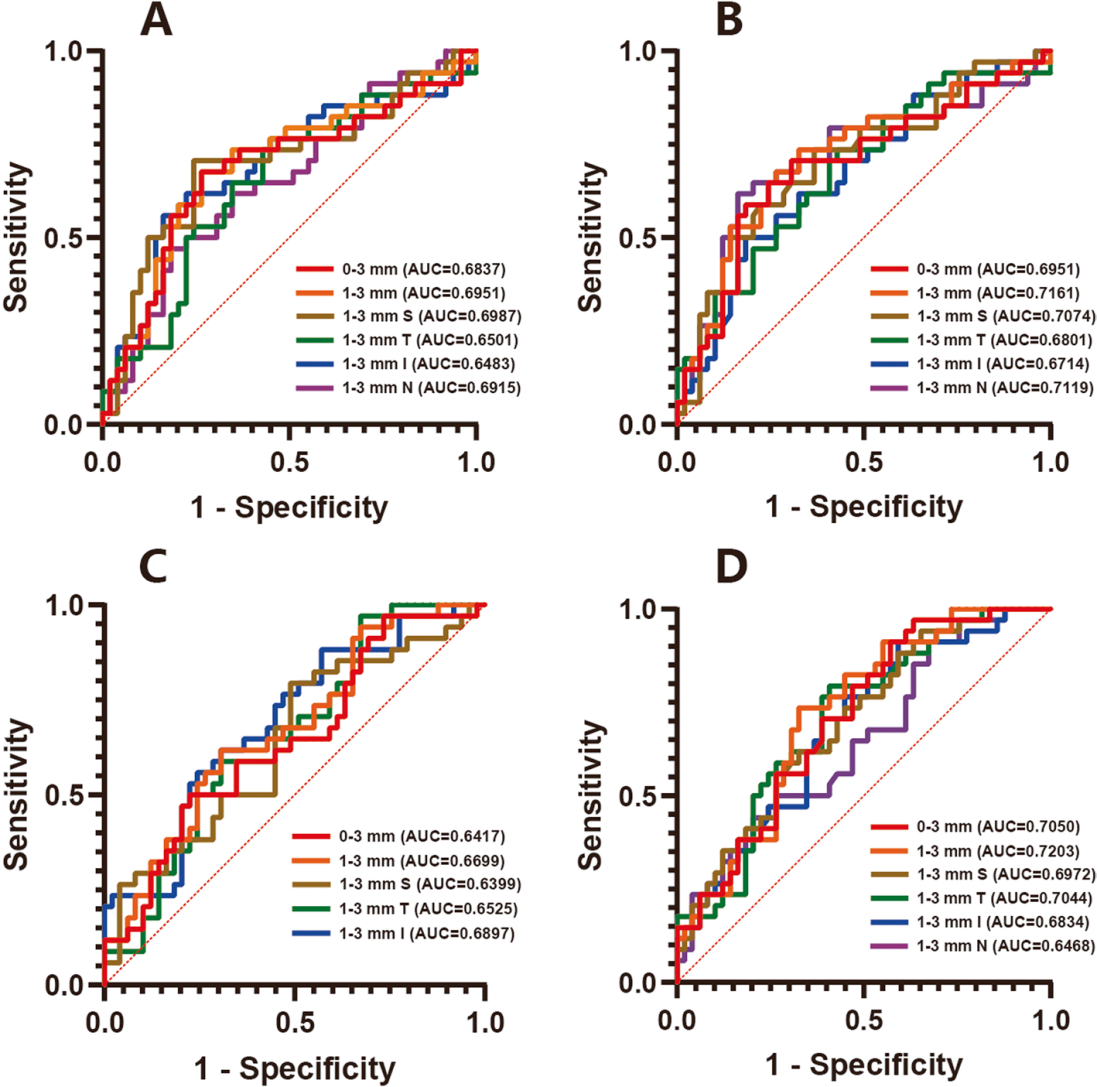
ROC curve of vessel density and flow area to distinguish AD from HC. **A.** Vessel density of the SCP; **B.** Flow area of the SCP; **C.** Vessel density of the ICP; and **D.** Flow area of the ICP

For efficiency of every single optical parameter, to differentiate AD patients from HC, both vascular density and flow area in the SCP and ICP displayed low to moderate diagnostic value separately in almost all quadrants. The flow area performed slightly better than the vascular density, with the AUC of the former ranging from 0.6714 to 0.7161 in the SCP (*p* ranged from 0.0009 to 0.0082) and 0.6468 to 0.7203 in the ICP (*p* ranged from 0.0007 to 0.0236), while the AUC of the latter ranged from 0.6483 to 0.6987 in the SCP (*p* ranged from 0.0026 to 0.0222) and 0.6399 to 0.6897 in the ICP (*p* ranged from 0.0034 to 0.0310) (Fig. 3). Neither vascular density nor flow area in the 0–1 mm circle showed any capability for differentiation.

Although the PCA group displayed a significant difference from the HC group in flow area and vessel density of the SCP, only the flow area of five quadrants in the ICP showed a low to moderate efficiency in distinguishing PCA patients from HC: 0–3 mm circle (AUC = 0.7132, *p* = 0.0078), 1–3 mm ring (AUC = 0.6950, *p* = 0.0150), SI (AUC = 0.6933, *p* = 0.0159), II (AUC = 0.7086, *p* = 0.0092), and NI (AUC = 0.6661, *p* = 0.0382) (Supplementary Fig. 1). No individual indicator was able to distinguish between the AD and PCA groups.

**Fig. 4:**
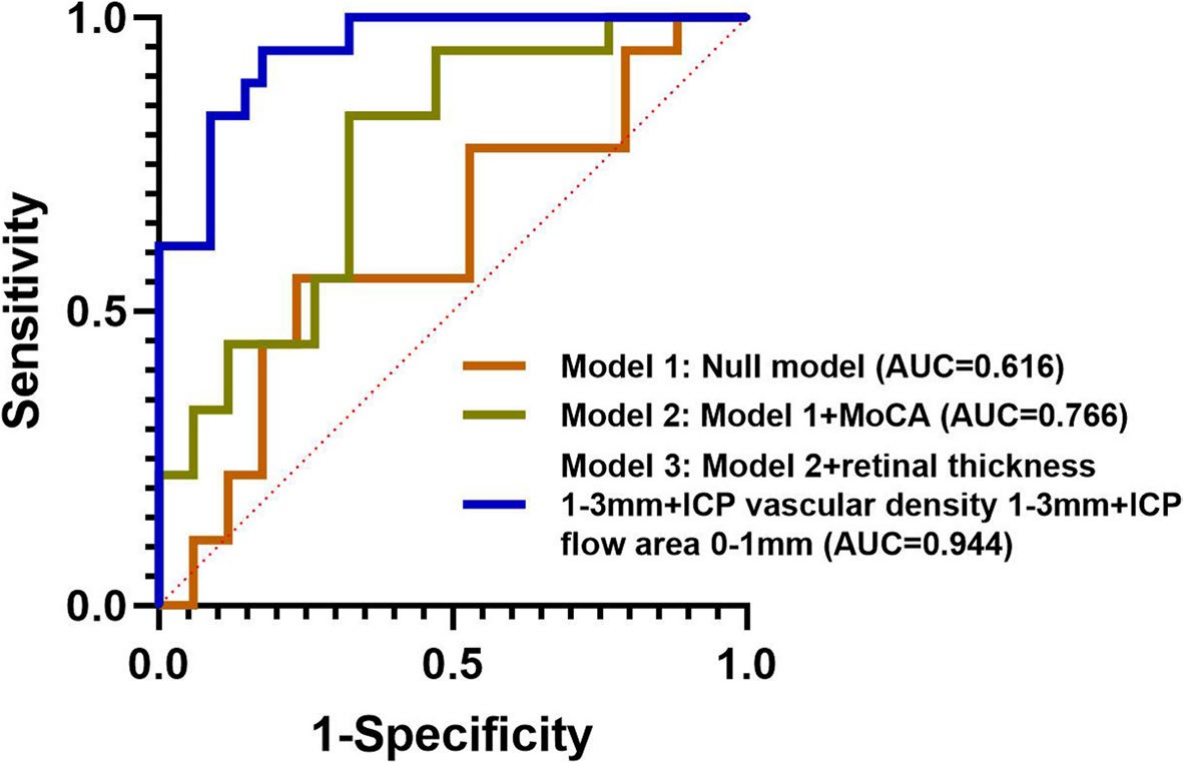
ROC curve of multivariate Logistic regression models of identifying PCA from AD. Confounding demographic data as age, sex proportion and years of education was used as independent factors in model 1 (null model). MoCA scores were added as clinical data into the null model to get model 2. Retinal thickness and vascular density of ICP in the 1-3 mm ring, with flow area of ICP in the 0-1 mm circle were added as optical parameters into the model 2 to get model 3

For multivariable diagnostic models, confounding demographic data as age, sex proportion and years of education was used as independent factors in all models. MMSE performed better than any combination of optical parameters in identifying AD and PCA from HC (AUC = 1, *p* < 0.001). Flow area of SCP in 1–3 mm ring was the best optical predictor for AD out of HC (AUC = 0.768, *p* < 0.001), as flow area of SCP in 0–1 mm circle and 1–3 mm ring was the best for PCA out of HC (AUC = 0.898, *p* < 0.001). In addition, PCA could be distinguished from AD using the optimal combination of MoCA, retinal thickness and vascular density of ICP in the 1–3 mm ring, with flow area of ICP in the 0–1 mm circle (AUC = 0.944, *p* < 0.001) (Fig. 4).

## Discussion

Retinal thickness, particularly of the RNFL and GCL, has been the focus of researchers studying optical changes in AD or other neurodegenerative diseases: a meta-analysis that included 126 papers before January 2021 showed that the thickness of the peripapillary RNFL, total macular, and subfoveal choroid was significantly reduced in AD patients compared with HC [25]. However, similar results were not observed in the present study, probably because AD patients with different phases of disease development were included. Salobrar-García et al. showed that reduced RNFL thickness was a dominant ocular characteristic in early AD, whereas intermediate and late AD patients could present with increased RNFL thickness [26]. Unexpectedly, a thinning compared with the HC group was observed in several quadrants of the RNFL, GCL + IPL, and INL in the PCA group, where the 0–3 mm circle and the NI area appeared to be more sensitive than other quadrants. Because of the limited number of studies on the ocular structures of PCA patients, conclusions about the impaired anterior visual pathway and the association of significantly altered quadrants with impaired brain regions in PCA patients need to be made with greater caution.

To our knowledge, there have been no reports of increased choroidal thickness in PCA patients. For AD patients, the existing studies frequently have reported a decrease in choroidal thickness [26–29]. Pathologic and simple high myopia are two common causes of reduced choroidal thickness, which are also closely associated with decreased choroidal blood perfusion [30]. In the present study, participants with high myopia were excluded, and diopters were matched between the three groups. Valuable data on choroidal perfusion were not obtained owing to software processing limitations. Therefore, the clinical value of the result that the PCA group had a thicker choroid than the AD group in the 0–1 mm circle and TI regions needs to be further verified.

A meta-analysis that included nine studies showed that the FAZ area tended to be larger in AD patients, without reaching statistical significance [31]. An increase of FAZ area was thought to be a predictor of preclinical AD [19, 20], which could result from a retinal co-degeneration with the brain due to β-amyloid accumulation in the cortex [32, 33] or a primitive accumulation of β-amyloid in the retina [34, 35]. No difference in the FAZ area was found among the three groups, which was consistent with the results of some previous studies [36, 37]. Because no corroboration of neuropathologic or local retinal pathologic findings was obtained in this study, none of the explanations for this result are sufficiently convincing.

The flow area and vessel density of the SCP and ICP in both the AD and PCA groups were significantly decreased in most of the ETDRS ring regions compared with the HC group, with more significant differences in the AD group than in the PCA group and more in the SCP than ICP, which is consistent with some previous reports [38, 39]. The ROC curves also reflected the value of the vascular density and flow area of the SCP and ICP in discriminating between the HC and AD patients; the expected diagnostic efficacy was not observed in the PCA group, perhaps because of the unbalanced number of subjects compared with the HC group. PCA patients could share a similar low perfusion of the microvascular network as AD patients. However, Zabel et al. emphasized that AD patients displayed a decreasing trend of the flow area and vessel density in the DCP rather than in the superficial vessels [40]. In addition, one article reported instead increased retinal perfusion in preclinical AD [41], reminding investigators that they should attempt to complete AD biomarker testing to avoid bias when conducting cross-sectional studies.

The results of multivariable logistic regression suggested that MMSE has an absolute advantage in distinguishing patients with AD or PCA from healthy people, which is consistent with our clinical experience, as the flow area of SCP played an auxiliary role in the identification. MoCA is more efficient than MMSE in distinguishing patients with AD and PCA, which may be resulted from higher visual participation requirement in MoCA. In this study, there was no significant difference in CDR and MMSE between the AD group and the PCA group, but the MoCA scores of PCA patients were significantly lower than that of the AD patients. This also reminds us that scales requiring more visual involvement can amplify the cognitive impairment of PCA and help differentiate PCA from typical AD patients [1, 42].

Most studies have analyzed the correlation between retinal structure (e.g., the RNFL, GCL, etc.) thickness, and cognitive level (e.g., MMSE, MoCA scores), and the results were either correlated [17, 43] or uncorrelated [44, 45]. The present study investigated the correlation between the retinal vascular plexus and cognitive level, where vascular density and flow area in the ICP displayed a relatively strong, although still unsatisfying, correlation. The result may suggest that altered vascular structure was not a major cause of cognitive impairment in AD or PCA.

Overall, this study filled a gap in the data related to fundus structure and vascular perfusion in patients with PCA. Some of the differences between the groups found in the study cannot be convincingly explained at this stage and need to be supported by greater data and more in-depth studies. There were some limitations in this study. First, AD and PCA diagnoses were based on clinical and neuroimaging features, only partial patients received cerebrospinal fluid biomarkers and/or pathological markers based on the PET technique, which may lead to an inaccurate diagnosis or an incorrect inclusion of patients with superposition syndromes. Second, the overall sample size was small, particularly the PCA group, which may minimize the real effect. Third, owing to severe eccentricity in most eyes, images in the range of 3–6 mm were discarded for the parameter measurement to avoid obtaining false results, which was not in agreement with the recommended "minimum data set" by the Atlas of Retinal Imaging in Alzheimer's Study [46]. Finally, an investigation for retinal pathological markers of PCA and AD was not performed, which may have resulted in overlooking the underlying mode of onset and pathogenic mechanism.

## Conclusions

This study investigated alternations of retinal structural and vascular indicators in PCA patients and validated these alternations in AD patients. The SS-OCTA technique may be helpful for non-invasive identification of AD and PCA from HC, and a promising method of differentiating PCA from typical AD.

### Supplementary Information

**Supplementary Material 1.**

## Data Availability

Anonymous data used in this study are available to the public with reasonable request through appropriate data sharing protocols.

## Abbreviations

AD: Alzheimer’s disease
AUC: Area under curve
CDR: Clinical dementia rating scale
CNS: Central nervous system
DCP: Deep capillary plexus
ETDRS: Early Treatment Diabetic Retinopathy Study
FAZ: Foveal avascular zone
GCL: Ganglion cell layer
HC: Healthy controls
ICP: Intermediate capillary plexus
II: Inferior inner
INL: Inner nuclear layer
IPL: Inner plexiform layer
MMSE: Mini-mental State Examination
MoCA: Montreal Cognitive Assessment
NI: Nasal inner
OCT: Optical coherence tomography
OCTA: Optical coherence tomography angiography
OD: Oculus dexter
OS: Oculus sinister
PCA: Posterior cortical atrophy
RNFL: Retinal nerve fiber layer
ROC: Receiver operating characteristic curve
SCP: Superficial capillary plexus
SD: Standard deviation
SI: Superior inner
SS-OCT: Swept-source optical coherence tomography
TI: Temporal inner
