# Supplementary material for: Potential ocular indicators to distinguish posterior cortical atrophy and typical Alzheimer’s disease: a cross-section study using optical coherence tomography angiography
Source: Alzheimers Res Ther. 2024 Mar 25;16:64. doi: 10.1186/s13195-024-01431-w (PMC10962115; doi:10.1186/s13195-024-01431-w)
Supplement: Supplementary file 1 — Supplementary Material 1. [file 13195_2024_1431_MOESM1_ESM.docx]

**Supplementary materials**

**Supplementary Table 1** Comparison of thickness of choroid in three groups

**Supplementary Table 2** Comparison of thickness of retina in three groups

**Supplementary Table 3** Comparison of thickness of ganglion cell layer (GCL) + inner plexiform layer (IPL) in three groups

**Supplementary Table 4** Comparison of thickness of inner nuclear layer (INL) in three groups

**Supplementary Table 5** Comparison of thickness of retinal nerve fiber layer (RNFL) in three groups

**Supplementary Table 6** Comparison of thickness of RNFL+GCL+IPL in three groups

**Supplementary Table 7** Comparison of vessel density of deep capillary plexus (DCP) in three groups

**Supplementary Table 8** Comparison of flow area of deep capillary plexus (DCP) in three groups

**Supplementary Table 9** Comparison of assessment of foveal avascular zone (FAZ) in three groups

**Supplementary Table 10** Correlation between capillary density, flow area of SCP and ICP with memory and visuospatial ability domain in MMSE

**Supplementary Figure 1** ROC curve of flow area in ICP to distinguish PCA from HC

**Supplementary Table 1** Comparison of thickness of choroid in three groups

|  | **HC (n=49)** |  | **AD (n=34)** |  | **PCA (n=18)** |
| --- | --- | --- | --- | --- | --- |
| 0-1 mm circle (μm) | 299.66±79.76 |  | 289.91±89.59 |  | 353.67±89.50^*^ |
| 0-3 mm circle (μm) | 294.05±81.34 |  | 285.92±88.63 |  | 344.05±83.10 |
| 1-3 mm ring (μm) | 293.35±81.76 |  | 285.42±88.63 |  | 342.84±82.75 |
| Superior inner (μm) | 305.99±86.60 |  | 298.67±89.67 |  | 356.34±75.38 |
| Temporal inner (μm) | 299.97±72.67 |  | 285.11±83.72 |  | 349.86±85.13^*^ |
| Inferior inner (μm) | 291.16±85.90 |  | 279.66±95.44 |  | 331.75±90.78 |
| Nasal inner (μm) | 276.31±93.95 |  | 278.29±92.72 |  | 333.44±89.12 |

^*^ *p* < 0.05: AD vs PCA, by *post hoc* test.

**Abbreviations:** HC healthy control, AD Alzheimer’s disease, PCA posterior cortical atrophy. Assessment values are mean ± standard deviation (SD).

**Supplementary Table 2** Comparison of thickness of retina in three groups

|  | **HC (n=49)** |  | **AD (n=34)** |  | **PCA (n=18)** |
| --- | --- | --- | --- | --- | --- |
| 0-1 mm circle (μm) | 255.36±22.48 |  | 253.69±17.56 |  | 251.82±22.57 |
| 0-3 mm circle (μm) | 321.29±13.32 |  | 317.75±14.64 |  | 315.97±12.32^*^ |
| 1-3 mm ring (μm) | 329.55±13.42 |  | 325.78±16.34 |  | 324.02±11.82 |
| Superior inner (μm) | 334.06±13.94 |  | 329.78±16.15 |  | 328.43±10.84 |
| Temporal inner (μm) | 320.04±13.37 |  | 316.90±16.38 |  | 315.93±12.83 |
| Inferior inner (μm) | 328.74±13.96 |  | 326.45±18.23 |  | 325.40±11.73 |
| Nasal inner (μm) | 335.39±14.25 |  | 329.99±16.18 |  | 326.32±14.63^**^ |

^*^ *p* < 0.05: HC vs PCA, by *post hoc* test.

^**^ *p* < 0.01: HC vs PCA, by *post hoc* test.

**Abbreviations:** HC healthy control, AD Alzheimer’s disease, PCA posterior cortical atrophy. Assessment values are mean ± standard deviation (SD).

**Supplementary Table 3** Comparison of thickness of ganglion cell layer (GCL) + inner plexiform layer (IPL) in three groups

|  | **HC (n=49)** |  | **AD (n=34)** |  | **PCA (n=18)** |
| --- | --- | --- | --- | --- | --- |
| 0-1 mm circle (μm) | 23.82±9.25 |  | 26.72±12.93 |  | 22.80±7.18 |
| 0-3 mm circle (μm) | 79.56±5.62 |  | 79.37±4.89 |  | 76.91±9.17^**^ |
| 1-3 mm ring (μm) | 86.54±5.90 |  | 85.97±4.84 |  | 83.69±9.72^**^ |
| Superior inner (μm) | 88.07±5.96 |  | 86.98±4.82 |  | 85.02±8.31* |
| Temporal inner (μm) | 83.66±6.52 |  | 83.95±4.52 |  | 81.82±10.03 |
| Inferior inner (μm) | 86.49±5.89 |  | 86.62±5.49 |  | 84.47±9.27 |
| Nasal inner (μm) | 87.69±6.34 |  | 86.34±5.86 |  | 83.47±12.13^**^ |

^*^ *p* < 0.05: HC vs PCA, by *post hoc* test.

^**^ *p* < 0.01: HC vs PCA, by *post hoc* test.

**Abbreviations:** HC healthy control, AD Alzheimer’s disease, PCA posterior cortical atrophy. Assessment values are mean ± standard deviation (SD).

**Supplementary Table 4** Comparison of thickness of inner nuclear layer (INL) in three groups

|  | **HC (n=49)** |  | **AD (n=34)** |  | **PCA (n=18)** |
| --- | --- | --- | --- | --- | --- |
| 0-1 mm circle (μm) | 26.16±4.51 |  | 26.51±4.12 |  | 26.38±4.00 |
| 0-3 mm circle (μm) | 43.15±3.14 |  | 42.78±2.67 |  | 42.45±2.39 |
| 1-3 mm ring (μm) | 45.28±3.19 |  | 44.82±3.01 |  | 44.47±2.35 |
| Superior inner (μm) | 46.46±3.51 |  | 45.59±3.68 |  | 45.69±2.74 |
| Temporal inner (μm) | 42.39±2.92 |  | 42.33±2.83 |  | 41.98±2.45 |
| Inferior inner (μm) | 45.18±3.17 |  | 45.42±3.12 |  | 44.84±2.80 |
| Nasal inner (μm) | 47.07±4.06 |  | 45.96±3.34 |  | 45.38±2.84^*^ |

^*^ *p* < 0.05: HC vs PCA, by *post hoc* test.

**Abbreviations:** HC healthy control, AD Alzheimer’s disease, PCA posterior cortical atrophy. Assessment values are mean ± standard deviation (SD).

**Supplementary Table 5** Comparison of thickness of retinal nerve fiber layer (RNFL) in three groups

|  | **HC (n=49)** |  | **AD (n=34)** |  | **PCA (n=18)** |
| --- | --- | --- | --- | --- | --- |
| 0-1 mm circle (μm) | 13.48±0.84 |  | 13.34±1.65 |  | 13.11±1.18 |
| 0-3 mm circle (μm) | 24.66±2.21 |  | 24.00±2.39 |  | 23.86±3.40^*^ |
| 1-3 mm ring (μm) | 26.06±2.77 |  | 25.34±2.55 |  | 25.21±3.77^*^ |
| Superior inner (μm) | 29.57±3.54 |  | 28.54±3.27 |  | 28.55±4.14 |
| Temporal inner (μm) | 20.02±1.94 |  | 19.55±1.85 |  | 19.37±2.25 |
| Inferior inner (μm) | 29.62±3.56 |  | 29.13±3.21 |  | 29.26±5.42 |
| Nasal inner (μm) | 25.03±2.86 |  | 24.12±2.75 |  | 23.68±3.77^**^ |

^*^ *p* < 0.05: HC vs PCA, by *post hoc* test.

^**^ *p* < 0.01: HC vs PCA, by *post hoc* test.

**Abbreviations:** HC healthy control, AD Alzheimer’s disease, PCA posterior cortical atrophy. Assessment values are mean ± standard deviation (SD).

**Supplementary Table 6** Comparison of thickness of RNFL+GCL+IPL in three groups

|  | **HC (n=49)** |  | **AD (n=34)** |  | **PCA (n=18)** |
| --- | --- | --- | --- | --- | --- |
| 0-1 mm circle (μm) | 37.30±9.68 |  | 40.06±14.39 |  | 35.91±7.64 |
| 0-3 mm circle (μm) | 104.21±7.72 |  | 103.37±7.05 |  | 100.77±12.50 |
| 1-3 mm ring (μm) | 112.60±8.22 |  | 111.31±7.08 |  | 108.91±13.41 |
| Superior inner (μm) | 117.65±8.95 |  | 115.52±7.39 |  | 113.57±12.26 |
| Temporal inner (μm) | 103.67±8.08 |  | 103.50±6.07 |  | 101.19±12.11 |
| Inferior inner (μm) | 116.11±8.74 |  | 115.76±8.33 |  | 113.73±14.59 |
| Nasal inner (μm) | 112.99±8.66 |  | 110.47±8.10 |  | 107.15±15.76 |

**Abbreviations:** HC healthy control, AD Alzheimer’s disease, PCA posterior cortical atrophy. Assessment values are mean ± standard deviation (SD).

**Supplementary Table 7** Comparison of vessel density of deep capillary plexus (DCP) in three groups

|  | **HC (n=49)** |  | **AD (n=34)** |  | **PCA (n=18)** |
| --- | --- | --- | --- | --- | --- |
| 0-1 mm circle (%) | 2.1493±3.1592 |  | 1.4586±1.9716 |  | 2.6585±1.7930 |
| 0-3 mm circle (%) | 6.8626±4.6093 |  | 6.5027±3.8769 |  | 8.9554±6.3452 |
| 1-3 mm ring (%) | 7.4534±4.8578 |  | 7.1350±4.2016 |  | 9.7450±7.0353 |
| Superior inner (%) | 7.7323±5.6086 |  | 7.6944±5.1464 |  | 10.2197±6.8880 |
| Temporal inner (%) | 8.0702±5.7828 |  | 7.0440±4.9525 |  | 9.9436±7.7846 |
| Inferior inner (%) | 7.6422±4.9815 |  | 6.9306±5.4031 |  | 9.7181±7.8872 |
| Nasal inner (%) | 6.3717±5.0130 |  | 6.8739±5.3659 |  | 9.1012±7.0657 |

**Abbreviations:** HC healthy control, AD Alzheimer’s disease, PCA posterior cortical atrophy. Assessment values are mean ± standard deviation (SD).

**Supplementary Table 8** Comparison of flow area of deep capillary plexus (DCP) in three groups

|  | **HC (n=49)** |  | **AD (n=34)** |  | **PCA (n=18)** |
| --- | --- | --- | --- | --- | --- |
| 0-1 mm circle (mm^2^) | 0.0643±0.0304 |  | 0.0553±0.0284 |  | 0.0577±0.0223 |
| 0-3 mm circle (mm^2^) | 1.5586±0.2101 |  | 1.4282±0.3245 |  | 1.5429±0.2192 |
| 1-3 mm ring (mm^2^) | 1.4943±0.1882 |  | 1.3730±0.3054 |  | 1.4852±0.2135 |
| Superior inner (mm^2^) | 0.3827±0.0513 |  | 0.3539±0.0871 |  | 0.3841±0.0554 |
| Temporal inner (mm^2^) | 0.3685±0.0537 |  | 0.3263±0.0867^*^ |  | 0.3592±0.0573 |
| Inferior inner (mm^2^) | 0.3809±0.0493 |  | 0.3532±0.0791 |  | 0.3772±0.0527 |
| Nasal inner (mm^2^) | 0.3621±0.0461 |  | 0.3396±0.0752 |  | 0.3647±0.0596 |

^*^ *p* < 0.05: HC vs AD, by *post hoc* test.

**Abbreviations:** HC healthy control, AD Alzheimer’s disease, PCA posterior cortical atrophy. Assessment values are mean ± standard deviation (SD).

**Supplementary Table 9** Comparison of assessment of foveal avascular zone (FAZ) in three groups

|  | **HC (n=50)** |  | **AD (n=30)** |  | **PCA (n=20)** |
| --- | --- | --- | --- | --- | --- |
| FAZ area (mm^2^) | 0.4144±0.1094 |  | 0.3995±0.1278 |  | 0.3948±0.1239 |
| Perimeter (mm) | 2.718±0.3679 |  | 2.684±0.3852 |  | 2.622±0.4205 |
| CI | 0.6969±0.0736 |  | 0.6805±0.0779 |  | 0.7064±0.0578 |
| FD | 41.49±3.44 |  | 39.54±4.34 |  | 40.50±4.60 |

**Abbreviations:** HC healthy control, AD Alzheimer’s disease, PCA posterior cortical atrophy, FAZ foveal avascular zone, CI circularity index, FD fractal dimension. Assessment Values are mean ± standard deviation (SD).

**Supplementary Table 10** Correlation between capillary density, flow area of SCP and ICP with memory and visuospatial domain in MMSE

|  | **SCP** | | | | |  | **ICP** | | | | |
| --- | --- | --- | --- | --- | --- | --- | --- | --- | --- | --- | --- |
|  | **Vessel density** | |  | **Flow area** | |  | **Vessel density** | |  | **Flow area** | |
|  | *r* | *p* |  | *r* | *p* |  | *r* | *p* |  | *r* | *p* |
| **Memory domain^1^** | | | | | | | | | | | |
| 0-1 mm circle | 0.0671 | 0.5048 |  | 0.0797 | 0.4282 |  | -0.0875 | 0.3845 |  | 0.0593 | 0.5557 |
| 0-3 mm circle | 0.0733 | 0.4662 |  | 0.1447 | 0.1488 |  | 0.0496 | 0.6221 |  | 0.1481 | 0.1394 |
| 1-3 mm ring | 0.0660 | 0.5121 |  | 0.1286 | 0.1999 |  | 0.0549 | 0.5857 |  | 0.1467 | 0.1432 |
| Superior inner | 0.1099 | 0.2738 |  | 0.1404 | 0.1614 |  | 0.0508 | 0.6137 |  | 0.1344 | 0.1803 |
| Temporal inner | 0.1122 | 0.2640 |  | 0.1234 | 0.2189 |  | -0.0602 | 0.5496 |  | 0.0581 | 0.0804 |
| Inferior inner | 0.0423 | 0.6745 |  | 0.0848 | 0.3989 |  | 0.0763 | 0.4485 |  | 0.1506 | 0.1327 |
| Nasal inner | 0.0162 | 0.8727 |  | 0.0861 | 0.3920 |  | -0.0276 | 0.7839 |  | 0.1171 | 0.2437 |
| **Visuospatial domain^2^** | | | | | | | | | | | |
| 0-1 mm circle | 0.1552 | 0.1212 |  | 0.1785 | 0.0741 |  | 0.1345 | 0.1800 |  | 0.1526 | 0.1277 |
| 0-3 mm circle | 0.0507 | 0.6135 |  | 0.1274 | 0.2044 |  | 0.1543 | 0.1233 |  | 0.2390 | **0.0161** |
| 1-3 mm ring | 0.0278 | 0.7825 |  | 0.0994 | 0.3225 |  | 0.1355 | 0.1767 |  | 0.2022 | **0.0426** |
| Superior inner | 0.0967 | 0.3358 |  | 0.1456 | 0.1464 |  | 0.0624 | 0.5354 |  | 0.0804 | 0.4239 |
| Temporal inner | 0.0450 | 0.6553 |  | 0.0567 | 0.5741 |  | -0.0048 | 0.9621 |  | 0.1932 | 0.4239 |
| Inferior inner | -0.0675 | 0.5024 |  | -0.0204 | 0.8395 |  | 0.1563 | 0.1186 |  | 0.2112 | **0.0340** |
| Nasal inner | -0.0320 | 0.7507 |  | 0.0502 | 0.6178 |  | 0.0326 | 0.7465 |  | 0.1890 | 0.0584 |

^1^ Memory domain refers to Registration and Recall parts in MMSE, maximum score for 6.

^2^ Visuospatial domain refers to sentence writing and picture copying parts in MMSE, maximum score for 2.

Bold type of *p* for statistical significance.


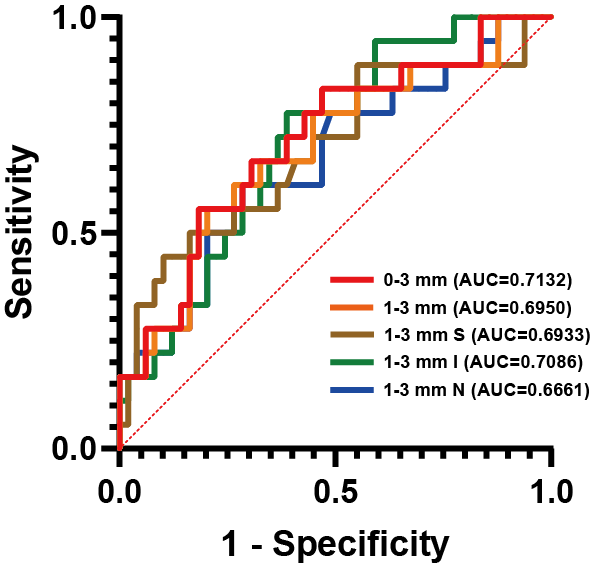


**Supplementary Figure 1** ROC curve of flow area in ICP to distinguish PCA from HC
